# Roles of specialized pro-resolving mediators and omega-3 polyunsaturated fatty acids in periodontal inflammation and impact on oral microbiota

**DOI:** 10.3389/froh.2023.1217088

**Published:** 2023-07-25

**Authors:** Chun-Teh Lee, Gena D. Tribble

**Affiliations:** Department of Periodontics and Dental Hygiene, School of Dentistry, University of Texas Health Science Center at Houston, Houston, TX, United States

**Keywords:** specialized pro-resolving mediators, lipid mediator, periodontitis, microbiome, host modulation, omega - 3 - fatty acid, host-pathogen interactions

## Abstract

Periodontitis is a chronic inflammatory disease induced by dysbiotic dental biofilms. Management of periodontitis is primarily anti-bacterial via mechanical removal of bacterial biofilm. The successful resolution requires wound healing and tissue regeneration, which are not always achieved with these traditional methods. The discovery of specialized pro-resolving mediators (SPMs), a class of lipid mediators that induce the resolution of inflammation and promote local tissue homeostasis, creates another option for the treatment of periodontitis and other diseases of chronic inflammation. In this mini-review, we discuss the host-modulatory effects of SPMs on periodontal tissues and changes in the taxonomic composition of the gut and oral microbiome in the presence of SPMs and SPM precursor lipids. Further research into the relationship between host SPM production and microbiome-SPM modification has the potential to unveil new diagnostic markers of inflammation and wound healing. Expanding this field may drive the discovery of microbial-derived bioactive therapeutics to modulate immune responses.

## Epidemiology of periodontitis

Oral health is an essential public health issue. Oral diseases cause pain, discomfort, masticatory malfunction, esthetically unacceptable appearance, and overall lower quality of life. Oral health is also highly associated with systemic health. Uncontrolled oral diseases can worsen systemic conditions. According to the 2022 World Health Organization (WHO) Global Oral Health Status Report, oral diseases are estimated to affect approximately 3.5 billion people worldwide, with 3 out of 4 affected people living in middle-income countries. The three most prevalent oral conditions are untreated caries, severe periodontitis, and extensive tooth loss (1). Among the prevalent oral diseases, periodontitis has one of the most complicated mechanisms of pathogenesis. Many researchers are studying its pathogenesis and developing novel diagnostic methods and therapy.

Periodontitis is a biofilm-induced chronic inflammatory disease characterized by gingival inflammation and alveolar bone loss around teeth. Untreated periodontitis leads to gingival bleeding, suppuration, pain, and progressive bone loss, eventually causing tooth loss. According to the 2009–2014 National Health and Nutrition Examination Survey (NHANES), in the United States, around 61 million adults over 30 years old have periodontitis (42.2%), with 7.8% having severe periodontitis (2). The global cost of lost productivity from severe periodontitis alone was $39 billion yearly, based on 2015 data (1). A recent report demonstrated that periodontal disease generally resulted in indirect and direct costs of $154.06 billion in the United States and €158.64 billion in Europe in 2018 (3).

## Pathogenesis of periodontitis

The disproportionate inflammatory host response and microbiota dysbiosis are the two major etiologic factors in the pathogenesis of periodontitis (4, 5). These two factors do not always exist simultaneously, but they influence each other. The coexistence of these two factors results in the development of periodontitis. A pathogenic biofilm is a prerequisite for periodontitis initiation. Several bacterial species, such as the core red complex species *Porphyromonas gingivalis (P. gingivalis)*, *Tannerella forsythia* (*T.forsythia*)*,* and *Treponema denticola* (*T.denticola*), are significantly associated with the severity and progression of periodontitis (6). Their virulence factors causing periodontal tissue destruction are also identified. However, these species are usually present in small amounts. The concept of keystone pathogens was proposed (7). The keystone pathogens, such as *P. gingivalis*, have a relatively small proportion of the overall microbial biomass but can dysregulate immune responses and induce microbiota dysbiosis, accelerating periodontal inflammation. This concept explains that the presence of specific species can be critical for the progression of periodontitis. While technologies advance, more and more species are identified as being associated with periodontitis and promoting disease progression. The antagonistic and synergistic interactions between multiple species as a network will be worth studying to elucidate the complexity of oral microbiota (8).

Although the biofilm is required to initiate periodontitis, the destruction of periodontal tissues in periodontitis are mainly caused by inflammation induced by bacteria (9). Persistent inflammation and tissue destruction create an environment leading to dysbiotic microbiota (5, 10). Local inflammation induced by periodontitis can further influence systemic immune responses. The status of periodontal health and systemic health condition are mutually affected (11). Studies demonstrated that periodontitis is associated with increased systemic levels of cytokines, such as interleukin-1α (12), interleukin-1β (12–14), interleukin-6 (IL-6) (12, 15, 16), C-reactive protein (CRP) (16, 17), interferon-γ and tumor necrosis factor-α (12, 13) in serum or plasma. Periodontal therapy can reduce systemic levels of inflammatory mediators, such as CRP (18, 19) and IL-6 (16, 20). Systemic level changes of cytokines in periodontitis patients indicate that periodontitis may play a role in the etiologic mechanisms of systemic inflammatory diseases, and inflammation drives the pathogenesis of periodontitis.

## Treating periodontitis through host-modulation

For decades, the standard treatment has been biofilm removal via mechanical debridement, such as scaling and root planing (SRP). However, this approach has limited effects in patients with aggressive forms of periodontitis associated with dysregulated immune response. To address this issue, host-modulation has been considered. Host-modulation therapy aims to modify the host response by reducing those damaging aspects of the inflammatory response leading to tissue destruction (21). Generally, there are two categories of host-modulation therapy: (1) modulating the host's inflammatory response by inhibition or resolution; (2) modulating the host's pathologic collagenolytic response in periodontal tissues (22). The first category includes the use of anti-inflammatory agents, such as non-steroidal anti-inflammatory drugs (NSAIDs), in addition to conventional periodontal treatment (e.g., SRP). The second category includes the use of subantimicrobial-dose doxycycline, which reduces collagenase activity to inhibit disease progression.

Restoration of a proper host immune response is crucial in treating periodontitis, but no effective host-modulatory approach currently exists. NSAIDs have been used to treat periodontitis, with positive clinical outcomes (23, 24). However, there are significant concerns regarding the long-term use of NSAIDs due to their adverse effects on the renal, cardiovascular, gastrointestinal, and hepatic systems (25). The clinical effects of anti-cytokine therapies used to treat rheumatoid arthritis and other immune diseases have also been investigated. Although these therapies can control inflammation, their clinical effects on periodontitis patients without immune disorders are not clear, and the adverse effects of systemic use, such as the increased risk of infection and malignancy, are concerning (21, 26). Although, statistically, subantimicrobial-dose doxycycline has beneficial clinical effects, but the absolute changes in pocket depth and clinical attachment level are limited. Also, the compliance with the long-term use can be challenging for patients (27). Host-modulation therapy for periodontitis is a promising approach, but more studies are required to make it practical and effective.

## Specialized pro-resolving mediators promote the resolution of inflammation and tissue regeneration

Anti-inflammation has been the central concept of treating periodontitis for years. Another option was presented with the discovery of specialized pro-resolving mediators (SPMs), a class of lipid mediators derived from omega-3 or omega-6 polyunsaturated fatty acids (PUFAs) that induce the resolution of inflammation and promote local tissue homeostasis (28). The resolution of inflammation is a proactive process induced by SPMs, including lipoxins, resolvins, protectins, and maresins. These SPMs are produced by enzymatic activation of membrane phospholipids and bind to specific G protein-coupled receptors on a variety of cells to regulate the immune response. In the resolution phase of inflammation, there are decreased infiltration of neutrophils, reduced levels of pro-inflammatory cytokines and lipid mediators, and increased recruitment of resolving macrophages, such as M2 macrophages, that clear the lesion by efferocytosis without immune suppression (29, 30). SPMs also stimulate the phagocytosis and killing of microbes (31). SPMs possess dual anti-inflammatory and pro-resolution properties.

Initial inflammation is required to defend against bacterial challenge. Neutrophils and macrophages play important roles in innate immunity. However, if acute inflammation is not properly resolved (e.g., excessive neutrophil infiltration and pro-inflammatory cytokine production), it leads to fibrosis, decreased apoptosis, impaired phagocytosis, and cellular senescence, resulting in chronic inflammation and tissue damage (32). Resolution of inflammation not only mitigates inflammation but also promotes tissue healing, regeneration, and reduction of pain. Due to the aforementioned characteristics, it is feasible to treat inflammatory diseases with SPMs. SPMs can control inflammation in many preclinical inflammatory-disease models, such as peritonitis (33), inflammatory bowel disease (34), diabetes (35), and periodontitis (36).

Recently, some newly identified conjugates of SPMs in tissue regeneration (CTRs) were identified, and these conjugates can promote tissue regeneration (30, 37). The novel cysteinyl-resolvin significantly accelerates tissue regeneration with planaria and inhibits human granuloma formation (37). A recent study demonstrated that porcine periodontal ligament stem cells (pPDLSCs) can synthesize cysteinyl-containing SPMs (cys-SPMs), specifically, maresin 3 conjugates in tissue regeneration (MCTR3), and pretreatment of pPDLSCs with MCTR3 reduced the production of acute and chronic proinflammatory cytokines and chemokines in an inflammatory environment (38).

## Specialized pro-resolving mediators in periodontitis

In periodontitis, preclinical studies have demonstrated that SPMs prevent and treat experimental periodontitis (36, 39). Topical application of RvE1 can prevent bone loss, regenerate the lost bone, change gene expression patterns in gingiva, and result in shifts of the oral microbiota (39, 40) and immune cellular components (41) in animals affected with experimental periodontitis. SPMs, including resolvins, lipoxins, and maresins, are now studied to understand their impact on periodontal inflammation and tissue healing. In the *in vitro* inflammatory condition, resolvin D1 (RvD1) can promote periodontal ligament fibroblasts (PDLF) proliferation (42, 43), reduce proinflammatory cytokine productions in gingival fibroblasts (44), and maresin-1 (MaR1) and resolvin E1 (RvE1) restore the regenerative properties of human PDLSCs (45, 46).

The impact of SPMs on periodontal pathogens was also investigated. An *in vitro* study showed that MaR1 enhanced intracellular antimicrobial reactive oxygen species production and restored impaired phagocytosis of *P.gingivalis* and *Aggregatibacter actinomycetemcomitans* (*A.actinomycetemcomitans)* in macrophages of localized aggressive periodontitis patients (47). This finding can be one of the mechanisms of oral microbiota shifts induced by SPMs.

For clinical applications, an effective vehicle to deliver SPMs is important to maintain high concentration and prevent lipid peroxidation. Membrane-shed vesicles, termed microparticles, have been used to deliver SPMs to treat experimental periodontitis (48, 49). Compared to SPM alone, SPM delivered in microparticles can increase treatment efficacy by targeting tissues without dilution or inactivation of the mediator. In a clinical trial, a formulated-mouthwash with methyl ester-benzo-lipoxin A4, one type of SPM, has been approved safe and could reduce local inflammation and increase the abundance of pro-resolution molecules in serum of human participants (46). Using SPMs to treat periodontitis in the clinic has the potential but more clinical studies are required.

## Lipid profiles and inflammatory diseases

**Figure 1:**
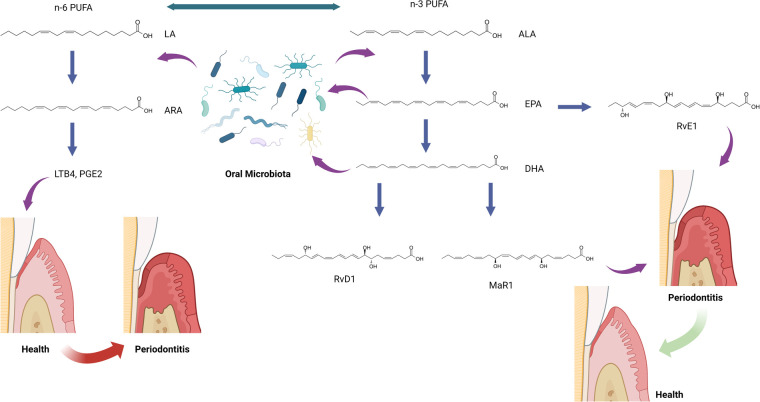
The hypothesis of oral microbiota and specialized pro-resolving mediator (SPM) interactions. ALA, alpha-linolenic acid; ARA, arachidonic acid; DHA, docosahexaenoic acid; EPA, eicosapentaenoic acid; LA, linoleic acid; LTB4, leukotriene B4; MaR1, maresin-1; n-3 PUFA, omega-3 polyunsaturated fatty acid; n-6 PUFA, omega-6 polyunsaturated fatty acid; PGE2, prostaglandin E2; RvD1, resolvin D1; RvE1, resolvin E1. This figure was created with BioRender.com.

Humans cannot efficiently produce the precursors for SPMs *de novo*. Instead, SPMs are derived from the ingestion of dietary omega-3 PUFA: alpha-linolenic acid (ALA), eicosapentaenoic acid (EPA), and docosahexaenoic acid (DHA) (Figure 1) (50). ALA, EPA, and DHA are incorporated as phospholipids into cellular membranes throughout the body, and SPMs are enzymatically released from these lipids to resolve inflammation (50). SPMs have been identified in many human samples, including milk (51), serum, lymphoid tissue (52), saliva, and gingival crevicular fluid (53, 54). These SPM levels have been shown to be involved in regulating the resolution of inflammation throughout the body, including the inflammatory status of mammary glands (51), the stability of atherosclerotic plaques (55), the severity of tuberculous meningitis (56), and the disease status of periodontitis (53, 54, 57). The action of omega-3 PUFA is in direct competition with dietary omega-6 PUFA, linoleic acid, which is the precursor to arachidonic acid (ARA), and the proinflammatory lipid mediators, prostaglandins and leukotrienes (58, 59). The relative ratios of dietary omega-3 and omega-6 PUFA are believed to contribute to homeostasis in the initiation and resolution of inflammation throughout the body (60, 61).

It is important to note that high doses of dietary PUFAs does not guarantee high production of SPMs. Actions of lipoxygenases are required to produce SPMs from PUFAs. Also, SPM corresponding receptors must be present on cells to bind SPMs inducing the resolution of inflammation.

## Effects of dietary lipids on Microbiota

As dietary compounds, omega-3 PUFAs are directly exposed to the microbiome of the oral cavity and digestive tract, and multiple studies in animals and humans describe significant changes in gut inflammation and the composition of the gut microbiome based on dietary PUFA quality and quantity (58, 62, 63). For example, in a comparison of mice fed with lard vs. fish oil (high in omega-3 PUFAs) as the major dietary fat, significant increases in *Lactobacilli, Bifidobacterium,* and *Akkermansia muciniphila* were detected in the fish oil diet, and transfer of *A. muciniphila* to the lard-fed mice could partially reduce the diet-induced intestinal inflammation and improve mucosal barrier function (64). The increase in *Bifidobacterium* in response to dietary EPA and DHA was confirmed in another mouse study, which also demonstrated that a diet high in omega-3 PUFAs from flax-seed and fish oil increases the bacterial diversity of the mouse gut (65). The same study determined that the flaxseed/fish oil diet increased the levels of ALA, EPA, and DHA in multiple tissues with a parallel decrease in ARA, indicating that the lipid membranes of host tissues are the ultimate destination for dietary omega-3 PUFAs. Lipids play important roles in microbial physiology; as structural components of the cell membrane, as energy storage modules, and for cell signaling and regulation of cellular activities (66). There is considerable overlap in lipid metabolic activities between eukaryotes and prokaryotes, providing opportunities for inter-kingdom cross-talk via lipid modification (58). In the gut microbiome, dietary lipids can be biotransformed by bacterial enzymes, resulting in downstream effects on host lipid physiology (58). *Bifidobacterium* and *Lactobacilli* are associated with reduced intestinal inflammation and diets high in omega-3 PUFAs. These bacteria have been shown to produce enzymes that modify omega-6 linoleic acid to a conjugated linoleic acid (CLA) that has anti-inflammatory effects and blocks the production of ARA-derived lipids (67). These same microorganisms produce a conjugated omega-3 alpha-linolenic acid (CLNA) with significant antioxidant effects (68).

These commensal bacteria utilize host lipids as an energy source and metabolize some lipids to new isoforms to enhance mucosal barrier function. Conversely, gut bacteria have also been associated with detrimental changes to dietary lipids, as demonstrated by studies of the microbiome associated with irritable bowel syndrome (69, 70). These studies begin to shed light on the potential circular relationship between dietary lipids, changes in the local host environment, and selection for bacteria that metabolize those same nutrients and benefit from changes to the environment (71). It is important to note that gut bacteria primarily influence the production of pro-inflammatory or pro-resolution lipids at the level of precursor molecules by manipulation of linoleic and alpha-linolenic acids. Identification of novel bioactive lipid molecules produced by gut bacteria has the potential to create new therapeutics for the regulation of the host immune system (72).

There are a few studies investigating the impact of omega-3 PUFAs on oral microbiota. An *in vitro* study showed that omega-3 PUFA, including EPA, DHA, and ALA, and their ester derivatives inhibited the growth of various oral bacteria, including *Streptococcus mutans*, *Candida albicans*, *A.actinomycetemcomitans*, *Fusobacterium nucleatum* (*F.nucleatum*), and *P*. *gingivalis* (73). The other *in vitro* study also showed that DHA and EPA possessed antibacterial activities against planktonic and biofilm forms of periodontal pathogens, *P.gingivalis* and *F.nucleatum* (74). High-dose omega-3 PUFA intake during non-surgical treatment in stage III or IV periodontitis patients was associated with reduced counts of periodontal pathogens, including *P.gingivalis*, *T.forsythia*, *T.denticola* and *A.actinomycetemcomitans* in a randomized clinical trial (75). The antimicrobial property of omega-3 PUFA indicates its potential effects on the composition of the oral microbiota. Potentially, gut and oral microbiota influenced by dietary lipids can affect each other (76, 77). More research is needed to investigate the impact of dietary lipids on oral microbiota and the interactions between oral and gut microbiota.

## Profiles of SPMs and Microbiota in periodontitis

The role of SPMs and relevant lipids in oral microbiota in periodontitis has been rarely investigated. Recently, SPMs, SPM pathway markers, and SPM corresponding receptor genes have been identified in human gingival tissues (57). A follow-up study aimed to analyze and integrate data on lipid mediator level (SPMs and SPM pathway markers), SPM receptor gene expression, and subgingival microbiome in subjects with periodontitis and healthy controls (78). The study included 13 periodontally healthy and 15 periodontitis subjects examined before or after non-surgical periodontal therapy. Gingival tissue and subgingival plaque samples were collected prior to and 8 weeks after non-surgical treatment, but these samples were only collected once in the healthy group before any prophylaxis. Correlations between lipid mediator levels, receptor gene expression, and bacterial abundance were analyzed using the Data Integration Analysis for Biomarker discovery using Latent components (DIABLO) and Sparse Partial Least Squares (SPLS) methods. The study demonstrated that specific bacterial species were significantly associated with lipid mediators in different inflammatory conditions. When comparing these correlated species in periodontitis before treatment to after treatment, a bacterial species, *Anaeroglobus geminatus*, was identified in both conditions and positively correlated with different lipid mediators. Both states (before and after treatment) had four lipid mediators, 5(S),12(S)-dihydroxy-6E,8Z,11E,14Z-eicosatetraenoic acid (5(S)12(S)-DiHETE), RvD1, MaR1, and leukotriene B4 (LTB4), correlated with different bacteria species. Among the nine bacterial species identified in the periodontitis after the SRP group, four *Selenomonas* species (*Selenomonas sp._oral_taxon_136*, *Selenomonas sp._oral_taxon_137*, *Selenomonas sp._oral_taxon_138*, *Selenomonas sp._oral_taxon_479*) were highly correlated with multiple lipid mediators. These identified bacteria are not considered periodontal pathogens in literature. Similar to the gut microbes described above, both *A. geminatus* and *Selenomonas spp.* encode enzymes capable of transforming linoleic and ALA-derived lipids, implying that they may play a similar role to gut bacteria in modifying oral lipids (79). It is also possible that the change in the local environment, including the inflammatory condition and lipid profiles, results in the presence of these bacteria, as we discussed in the other sections. These findings indicate the potential interactions between lipids, microbiota, and inflammation in periodontitis which has not been deeply investigated (Figure 1).

## Conclusion

These findings demonstrate that, similar to the influence of diet on the gut microbiome, the resolution of inflammation induced by SPMs is associated with shifts in the taxonomic composition of the oral microbiota. Potentially, SPMs and subgingival bacterial species may have interactions that open new possibilities for the identification of diagnostic biomarkers and the development of therapeutics for periodontitis.
