# Correction: Roles of specialized pro-resolving mediators and omega-3 polyunsaturated fatty acids in periodontal inflammation and impact on oral microbiota

**DOI:** 10.3389/froh.2025.1691158

**Published:** 2025-09-22

**Authors:** Chun-Teh Lee, Gena D. Tribble

**Affiliations:** Department of Periodontics and Dental Hygiene, School of Dentistry, University of Texas Health Science Center at Houston, Houston, TX, United States

**Keywords:** specialized pro-resolving mediators, lipid mediator, periodontitis, microbiome, host modulation, omega - 3 - fatty acid, host-pathogen interactions

In the published article, the Funding statement was missing. The correct Funding statement appears below.

FUNDING

This work was supported by the NIH/NIDCR grant R21DE031440.

The original article has been updated.

